# Simultaneous Genotyping of Three Nonsynonymous SNVs, rs1042602, rs1426654, and rs16891982 Involved in Skin Pigmentation by Fluorescent Probe-Based Melting Curve Analysis

**DOI:** 10.1155/humu/3468799

**Published:** 2025-07-23

**Authors:** Mikiko Soejima, Yoshiro Koda

**Affiliations:** Department of Forensic Medicine, Kurume University School of Medicine, Kurume, Japan

**Keywords:** melting curve genotyping, rs1042602, rs1426654, rs16891982, skin pigmentation

## Abstract

Three nonsynonymous single nucleotide variations (SNVs), rs1042602 in TYR (p.S192Y), rs1426654 in SLC24A5 (p.A111T), and rs16891982 in SLC45A2 (p.L374F), were associated with human skin pigmentation variation and may have recently undergone positive natural selection. Furthermore, these three SNVs have been reported to correlate with the risk and prognosis of melanoma. To simultaneously determine these three SNVs, a triplex fluorescent probe-based melting curve assay (FMCA) was developed. The method was validated by analyzing genomic DNA from subjects with known genotypes. For rs16891982, triplex FMCA did not allow good separation of genotypes with the initial polymerase enzyme mix used, but by changing the enzyme mix used, the three genotypes could be clearly distinguished. Using this method, we definitively genotyped these three SNVs in 93 European, 58 Tamil, 54 Sinhalese, and 52 Bangladeshi subjects. This method allows genotyping of rs1042602, rs1426654, and rs16891982 in a relatively large number of samples to perform association studies on skin pigmentation variation or melanoma risk.

## 1. Introduction

Three genes, the tyrosinase (TYR) gene, solute carrier family 24 member 5 gene (SLC24A5), and solute carrier family 45 member 2 gene (SLC45A2), have been reported to be implicated in human skin pigmentation [1–3]. Inactivating mutations in these genes have been reported to cause oculocutaneous albinism (OCA), that is, TYR mutations cause OCA1, SLC45A2 mutations cause OCA4, and SLC24A5 mutations cause OCA6 [4]. Furthermore, signatures of recent positive selection in European populations have been found around three nonsynonymous single nucleotide variations (SNVs) in three genes: rs1042602 in TYR (p.S192Y), rs1426654 in SLC24A5 (p.A111T), and rs16891982 in SLC45A2 (p.L374F) [5–7]. Almost no derived alleles of these three SNVs have been found in Africans and East and Southeast Asians. In rs1426654 and rs16891982 especially, the derived alleles are almost fixed in Europeans [5, 6, 8, 9], leading to a significantly large fixation index (*Fst,* the most frequently used measure of population differentiation among groups) between Europeans and other populations [10]. These three SNVs have been reported to act additively, which explains much of the natural variation in skin pigmentation in South Asian populations [1–3] and may also be useful in narrowing down potential suspects for unidentified individuals and perpetrators of crimes. In addition, they have been reported to be associated with melanoma risk and prognosis in several populations [11–13]. Therefore, it is useful to establish a simple and rapid method to genotype these three SNVs, preferably one that can genotype all of them in a single reaction.

TaqMan assays use dual-labeled fluorescent probes to enable quantitative DNA or RNA analysis and real-time PCR monitoring for SNV determination [14]. The 5⁣′ to 3⁣′ exonuclease activity of Taq polymerase cleaves the probe when it hybridizes to its complementary target, separating the fluorophore and quencher, and resulting in fluorescence [14]. Meanwhile, dual-labeled fluorescent probes are also used in fluorescence melting curve analysis (FMCA), which is one of the powerful methods for SNVs detection [15, 16]. For FMCA, any Taq polymerase with or without 5⁣′ to 3⁣′ exonuclease activity can be used, as there is no need to degrade the probe [16]. Compared to the TaqMan assay, FMCA has the advantage of being assayed with one probe per SNV, making it easier to perform multiplex assays.

In this study, we developed a method that can simultaneously determine the genotypes of rs1042602, rs1426654, and rs16891982 using triplex FMCA.

## 2. Materials and Methods

### 2.1. DNA Samples

We used anonymized genomic DNA from 58 Tamils, 54 Sinhalese, 52 Bangladeshi, and two Japanese subjects in this study [9, 17]. In addition, genomic DNA from 93 of 100 Europeans, the Human Variation Panel—Caucasian panel of 100 (Set 1), was purchased from the Coriell Institute for Medical Research (Camden, New Jersey).

### 2.2. Probes and Primers

PCR primers (SLC24A5-F primer: 5⁣′-TTCATTTATGTTCAGCCCTTGGA-3⁣′, SLC24A5-R primer: 5⁣′-TTCAGGAGCTGAACTGCCC-3⁣′) and a FAM-black hole quencher 1 (BHQ1)-labeled probe (SLC24A5-probe: FAM-5⁣′-GATGTTGCAGGCACAACTTTCATGG-3⁣′-BHQ1, position of rs1426654 is underlined), PCR primers (SLC45A2-F primer: 5⁣′-ACATCCTTAGGAGAGAGAAAGACT-3⁣′, SLC45A2-R primer: 5⁣′-GAGGAGTCGAGGTTGGATGT-3⁣′) and a HEX-BHQ1-labeled probe (SLC45A2 probe: HEX-5⁣′-AAACACGGAGTTGATGCACAAGCCC-3⁣′-BHQ-1, the position of rs16891982 is underlined), and PCR primers (TYR-F primer: 5⁣′-TGACCTCTTTGTCTGGATGCA-3⁣′, TYR-R primer: 5⁣′-TTCCCACCGCAACAAGAAGA-3⁣′) and a Cy5-BHQ2-labeled probe (TYR probe: CY5-5⁣′-GCTTGGGGGATCTGAAATCTGGAGA-3⁣′-BHQ-2, the position of rs1042602 is underlined), were used to detect three SNVs, rs1426654, rs16891982, and rs1042602. The genomic DNA sequence of rs16891982 in this study is the reverse sequence of mRNA, so C corresponds to G and G corresponds to C in the cDNA. Therefore, in cDNA, G is the ancestral type, and C is the derived type, but in the genomic DNA of this study, C is the ancestral type, and G is the derived type (light skin allele). All primers and probes were synthesized by Europhins Genomics (Tokyo, Japan).

### 2.3. Real-Time PCR Monitoring and FMCA

As previously reported [16], in FMCA using a dual-labeled fluorescence probe, the symmetric PCR did not give sufficient melting curve peaks, so it was necessary to perform the asymmetric PCR. Therefore, in this study, asymmetric PCR for FMCA was performed using a LightCycler 480 Instrument II (Roche Diagnostics, Tokyo, Japan) with a tenfold excess of reverse primers [18]. In this study, we used Premix Ex Taq (Probe qPCR) or Probe qPCR Mix MultiPlus (Takara Bio, Shiga, Japan). The 10-*μ*L PCR reaction mixture consisted of genomic DNA (1–10 ng), 5 *μ*L of a premixed solution, 50 nM of each F primer and 500 nM of R primer, and 200 nM of each fluorescent probe. The thermal profile for *Premix Ex Taq* (Probe qPCR) was an initial denaturation at 95°C for 30 s, followed by 45 cycles of denaturation at 95°C for 5 s, and annealing and extension at 60°C for 10 s, and that for Probe qPCR Mix MultiPlus was an initial denaturation at 95°C for 20 s, followed by 45 cycles of denaturation at 95°C for 1 s, and annealing and extension at 60°C for 20 s. For real-time PCR monitoring, FAM (465 to 510 nm), VIC/HEX/Yellow555 (533 to 580 nm), and Cy5/Cy5.5 filters (618 to 660 nm) were used to acquire fluorescent data at the end of each annealing and extension step.

After PCR amplification, samples were denatured at 95°C for 1 min, hybridized at 40°C for 1 min, and fluorescent data for FMCA were collected using FAM, VIC/HEX/Yellow555, and Cy5/Cy5.5 filters in the temperature range of 50°C to 80°C with a ramp rate of 0.10°C/s and 2 acquisitions/°C. Melting temperature (*Tm*) calculation and melting curve genotyping were performed using LightCycler 480 gene scanning software Version 1.5 (Roche Diagnostics) with the default setting.

## 3. Results and Discussion

**Figure 1:**
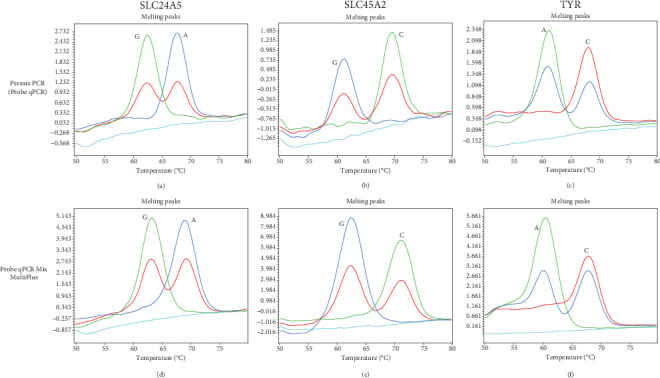
Melting curve genotyping of selected subjects by triplex FMCA. Results of (a) rs1426654, (b) rs16891982, and (c) rs1042602 using Premix Ex Taq (Probe qPCR). Results of (d) rs1426654, (e) rs16891982, and (f) rs1042602 using Probe qPCR Mix MultiPlus. Negative controls are indicated by light blue.

**Figure 2:**
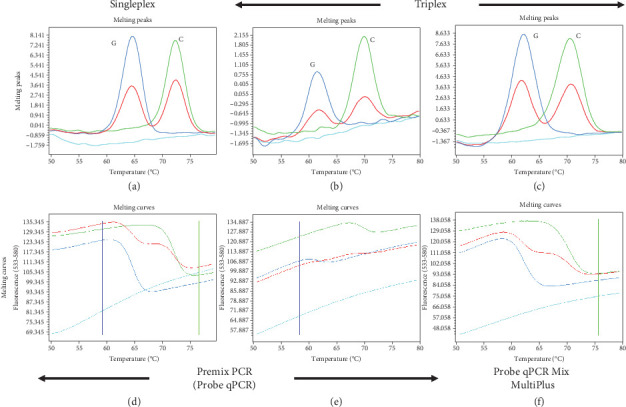
Improving results of FMCA of rs16891982 by changing the enzyme mix used. (a) Melting peak and (d) melting curve by singleplex FMCA using Premix Ex Taq (Probe qPCR). (b) Melting peak and (e) melting curve by triplex FMCA using Premix Ex Taq (Probe qPCR). (c) Melting peak and (f) melting curve for genotyping by triplex FMCA using Probe qPCR Mix MultiPlus. Negative controls are indicated by light blue.

In this study, we first attempted to simultaneously detect three SNVs, rs1042602 of TYR, rs1426654 of SLC24A5, and rs16891982 of SLC45A2, by triplex FMCA using Premix Ex Taq (Probe qPCR). For validation of genotyping by FMCA, we selected nine European subjects and two Japanese subjects whose rs1042602, rs1426654, and rs16891982 genotypes were confirmed by Sanger sequencing. Three genotypes of rs1042602 (C/C, *Tm*: about 68°C, C/A, *Tm*: about 60°C and 68°C, and A/A, *Tm*: about 60°C, A is a light skin allele) and three genotypes of rs1426654 (A/A, *Tm*: about 69°C, A/G, *Tm*: about 63°C and 69°C, and G/G, *Tm*: about 63°C, A is light skin allele) could each be clearly separated by triple FMCA (Figure 1a,c). Although the three genotypes of rs16891982 could be separated (C/C, *Tm*: about 71°C, C/G, *Tm*: about 62°C and 71°C, and G/G, *Tm*: about 62°C), the peak height was relatively low (Figures 1b and 2b,e), so the separation appeared to be somewhat poor when many samples were analyzed. On the other hand, the three genotypes of rs16891982 were clearly separated in singleplex FMCA due to sufficient peak height (Figure 2a,d), although the amplification signals were similar in singleplex and triplex FMCAs.

Next, we tried several DNA polymerase premixes to improve the resolution of triplex FMCA. Among them, qPCR Mix MultiPlus increased the FMCA peak height of not only rs16891982 but also rs1042602 and rs1426654 (Figures 1a, 1b, 1c, 1d, and 1f and Figures 2b, 2c, 2e, and 2f). As a result, genotyping results, especially for rs16891982, were significantly improved.

In the amplification step prior to multiplex FMCA, it is necessary to amplify gene fragments equally. Furthermore, in the subsequent FMCA, the salt concentration of the PCR mixture and the composition of additives involved in DNA formation are also important. We actually tried several heat-resistant DNA polymerase premixes, but most of them did not successfully amplify the product. Therefore, it was suggested that a mixture specialized for multiplex assays may be useful for performing multiplex FMCA. In this study, the shape of the melting curve exhibited unusual baseline behavior, rising rather than the typical decrease with increasing temperature. While the exact reason for this is unclear, it is presumed to be due to the secondary structure of the probe. Therefore, redesigning the probe again or using probes such as peptide nucleic acid (PNA) probe that increase *Tm* to shorten the length of the probe might help avoid the influence of the secondary structure [19]. We would like to explore these points further in the future.

Next, we determined the genotypes of rs1042602, rs1426654, and rs16891982 in 93 Caucasians, 58 Tamils, 54 Sinhalese, and 52 Bangladeshi using this method. As shown in Supporting Information 1–4 (Figures S1, S2, S3, and S4), genotyping was performed successfully. The frequencies of genotypes and their combinations in the three populations are shown in Table 1. The genotypes of rs1426654 and rs16891982 in 58 Tamils and 54 Sinhalese were consistent with previous genotyping results [9]. As expected, the frequencies of the three genotype combinations differed considerably between Europeans and South Asians. Although we cannot be certain due to the small sample size, there may be some regional differences among South Asians [2, 20].

As previously reported [21], in order to distinguish genotypes by melting curve analysis, each genotype was designed to have a *Tm* value interval of at least 1°C. Therefore, it was thought that the number of genotypes that could be accurately distinguished using *Tm* value for each color was limited. In fact, since there have been reports ranging from 9 to 12 using FMCA analysis with three fluorescent dyes in the virus genotyping method [22, 23], it is considered that the limitation for determining genotypes with a single fluorescent dye is between 3 and 4. In any case, the advantage of FMCA is that the number of genotypes that can be diagnosed is increased by using different fluorescent dyes.

## 4. Conclusions

We established a method to simultaneously determine the genotypes of three SNVs related to skin pigmentation variation. By using this method to examine the distribution of the three SNVs in a relatively large number of samples, it is possible to evaluate population admixtures and risk for melanoma, and analyze the gradient of the frequency of these genes related to latitude.

## Figures and Tables

**Table 1:** Genotype combinations of rs1426654, rs16891982, and rs1426654 in Caucasians, Tamils, Sinhalese, and Bangladeshi.

**Genotypes of rs1426654/rs16891982/rs1426654**	**Europeans**	**Tamils**	**Sinhalese**	**Bangladeshi**
AA/CC/AC	0	1	2	2
AA/CC/CC	0	5	13	13
AA/CG/AA	1	0	0	0
AA/CG/AC	6	0	0	0
AA/CG/CC	1	0	0	1
AA/GG/AA	10	0	0	0
AA/GG/AC	41	0	0	0
AA/GG/CC	31	0	0	0
AG/CC/AC	0	2	1	6
AG/CC/CC	0	21	24	18
AG/CG/AC	1	0	0	0
AG/CG/CC	0	0	0	3
AG/GG/AC	2	0	0	0
GG/CG/CC	0	1	1	0
GG/CC/AC	0	2	0	0
GG/CC/CC	0	25	13	8
GG/CG/CC	0	1	0	1
Total	93	58	54	52

## Data Availability

The data that support the findings of this study are available from the corresponding author upon reasonable request.

## Nomenclature

BHQ: black hole quencher
FMCA: fluorescence melting curve analysis
TYR: tyrosinase
SLC24A5: solute carrier family 24 member 5
SLC45A2: solute carrier family 45 member 2
*Tm*: melting temperature

## Supporting Information

Supporting Information 1 Figure S1: Melting curve genotyping of 93 European and one Japanese by triplex FMCA using Probe qPCR Mix MultiPlus. Genotyping results of (A) rs1426654, (B) rs16891982, and (C) rs1042602. Negative controls are indicated by light blue.

Supporting Information 2 Figure S2: Melting curve genotyping of 58 Tamil subjects by triplex FMCA using Probe qPCR Mix MultiPlus. Genotyping results of (A) rs1426654, (B) rs16891982, and (C) rs1042602. Negative controls are indicated by light blue.

Supporting Information 3 Figure S3: Melting curve genotyping of 54 Sinhalese subjects by triplex FMCA using Probe qPCR Mix MultiPlus. Genotyping results of (A) rs1426654, (B) rs16891982, and (C) rs1042602. Negative controls are indicated by light blue.

Supporting Information 4 Figure S4: Melting curve genotyping of 52 Bangladeshi subjects by triplex FMCA using Probe qPCR Mix MultiPlus. Genotyping results of (A) rs1426654, (B) rs16891982, and (C) rs1042602. Negative controls are indicated by light blue.
