# Supplementary material for: Simultaneous Genotyping of Three Nonsynonymous SNVs, rs1042602, rs1426654, and rs16891982 Involved in Skin Pigmentation by Fluorescent Probe-Based Melting Curve Analysis
Source: Hum Mutat. 2025 Jul 23;2025:3468799. doi: 10.1155/humu/3468799 (PMC12310321; doi:10.1155/humu/3468799)
Supplement: Supporting Information 2 — Figure S2: Melting curve genotyping of 58 Tamil subjects by triplex FMCA using Probe qPCR Mix MultiPlus. Genotyping results of (A) rs1426654, (B) rs16891982, and (C) rs1042602. Negative controls are indicated by light blue. [file 3468799.f2.pdf]

Supplementary Figure 2

A

SLC24A5

B

SLC45A2

C

TYR

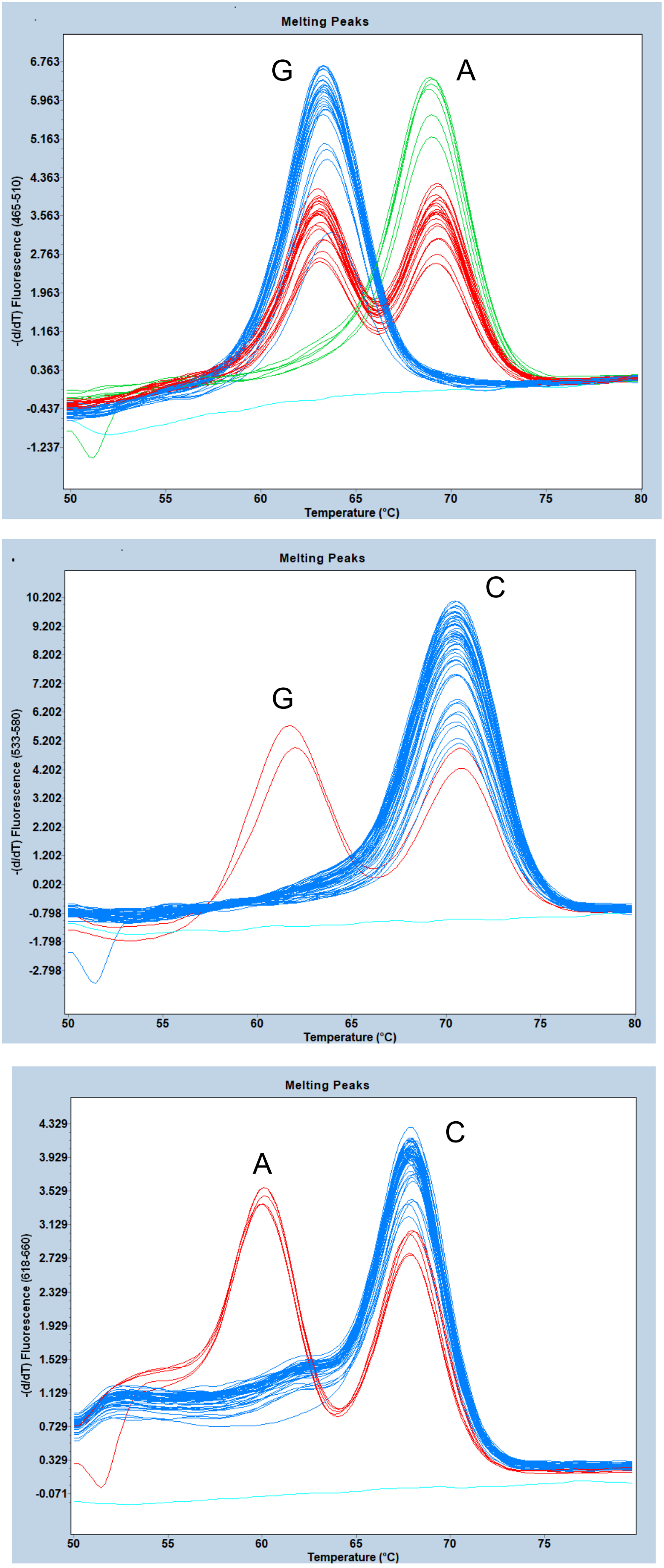

Supplementary Figure 2. Melting curve genotyping of 58 Tamil subjects by triplex FMCA using Probe qPCR Mix MultiPlus. Genotyping results of rs1426654 (A), rs16891982 (B), and rs1042602 (C). Negative contols are indicated by light blue.
